# Supplementary material for: University service utilization patterns in students with specific learning disabilities: An institutional cross-sectional study
Source: PLoS One. 2025 Jul 16;20(7):e0328350. doi: 10.1371/journal.pone.0328350 (PMC12266441; doi:10.1371/journal.pone.0328350)
Supplement: S1 Table — (PDF) [file pone.0328350.s001.pdf]

University Service Utilization Patterns in Students with Specific Learning Disabilities: An Institutional Cross-Sectional Study

S1 Table. Factors influencing the use of Common and Specific Services

Use of Common Services								
	B	S.E.	Wald	df	Sig.	Exp(B)	95% C.I.for EXP(B)	
							Lower	Upper
Age (years)	.273	.453	.365	1	.546	1.315	.541	3.194
Gender	-.523	.297	3.093	1	.079	.593	.331	1.062
Nationality	-.263	.291	.816	1	.366	.769	.435	1.359
Total family income (SAR)	-.311	.319	.950	1	.330	.732	.392	1.370
Affiliated Program	.279	.195	2.040	1	.153	1.322	.901	1.939
Academic Year	.254	.088	8.403	1	.004	1.290	1.086	1.531
cGPA	.319	.220	2.102	1	.147	1.376	.894	2.117
Self-declaration of educational outcome	.841	.371	5.124	1	.024	2.318	1.119	4.798
Self-assessment of health	-1.695	.901	3.536	1	.060	.184	.031	1.074
Chronic disease Status	-.614	.374	2.691	1	.101	.541	.260	1.127
Sleep status	.294	.275	1.145	1	.285	1.342	.783	2.300
Physical activity	.427	.295	2.091	1	.148	1.533	.859	2.734
BMI type	.000	.169	.000	1	.999	1.000	.718	1.393

Self-declared QOL	.101	.333	.091	1	.763	1.106	.575	2.126
Satisfaction with health	-.156	.317	.242	1	.623	.855	.459	1.594
Dyslexia	-.582	.468	1.549	1	.213	.559	.223	1.397
Dysgraphia	.330	.307	1.160	1	.282	1.391	.763	2.538
Dyscalculia	-.049	.308	.025	1	.874	.952	.521	1.742
Auditory processing disorder	-.013	.391	.001	1	.973	.987	.458	2.124
Language processing disorder	.395	.406	.950	1	.330	1.485	.670	3.289
Nonverbal learning disabilities	-.623	.398	2.450	1	.118	.536	.246	1.170
Visual perceptual/visual motor deficit	1.353	.384	12.415	1	.000	3.867	1.822	8.207
Constant	-1.434	1.787	.644	1	.422	.238		
**Use of specific Services**								
							95% C.I.for EXP(B)	
	B	S.E.	Wald	df	Sig.	Exp(B)	Lower	Upper
Age (years)	.600	.467	1.649	1	.199	1.822	.729	4.550
Gender	.728	.300	5.882	1	.015	2.070	1.150	3.727
Nationality	-.489	.290	2.846	1	.092	.614	.348	1.082
Total family income (SAR)	.066	.316	.043	1	.835	1.068	.575	1.983
Affiliated Program	.074	.196	.141	1	.707	1.076	.733	1.580
Academic Year	-.083	.087	.914	1	.339	.920	.777	1.091
cGPA	.362	.215	2.836	1	.092	1.436	.942	2.187

Self-declaration of educational outcome	.711	.364	3.807	1	.051	2.035	.997	4.156
Self-assessment of health	.483	.844	.328	1	.567	1.621	.310	8.473
Chronic disease Status	.146	.375	.152	1	.697	1.158	.555	2.415
Sleep status	-.772	.273	7.971	1	.005	.462	.271	.790
Physical activity	.112	.298	.142	1	.707	1.119	.624	2.007
BMI type	-.164	.170	.934	1	.334	.848	.608	1.184
Self-declared QOL	.267	.331	.650	1	.420	1.306	.683	2.497
Satisfaction with health	-.333	.320	1.083	1	.298	.717	.383	1.342
Dyslexia	1.004	.479	4.391	1	.036	2.728	1.067	6.976
Dysgraphia	.339	.309	1.203	1	.273	1.403	.766	2.570
Dyscalculia	-.322	.304	1.122	1	.290	.724	.399	1.315
Auditory processing disorder	.925	.390	5.629	1	.018	2.521	1.174	5.411
Language processing disorder	-.188	.393	.228	1	.633	.829	.384	1.790
Nonverbal learning disabilities	-.240	.388	.384	1	.536	.786	.368	1.682
Visual perceptual/visual motor deficit	-.326	.360	.822	1	.365	.722	.356	1.461
Constant	-3.316	1.807	3.366	1	.067	.036		
