# Supplementary material for: University service utilization patterns in students with specific learning disabilities: An institutional cross-sectional study
Source: PLoS One. 2025 Jul 16;20(7):e0328350. doi: 10.1371/journal.pone.0328350 (PMC12266441; doi:10.1371/journal.pone.0328350)
Supplement: S1 File — (PDF) [file pone.0328350.s002.pdf]

University Service Utilization Patterns in Students with Specific Learning Disabilities: An Institutional Cross-Sectional Study

Study Questionnaire

Sociodemographic characteristics

1. Age (years)
2. Gender
3. Education level of father
4. Education level of mother
5. Nationality
6. Employment status of father
7. Employment status of mother
8. Total family income (SAR per month)
9. Affiliated program
10. Academic year (including preparatory and internship years)
11. Grade Point Average (GPA) during the previous semester
12. Cumulative GPA
13. How would you rate your educational outcome?
14. Overall health self-assessment
15. Are you suffering from any chronic disease?
16. Sleep status
17. Physical activity
18. Height (cm)
19. Height (m)

20. Weight (kg)
21. How would you rate your quality of life?
22. How satisfied are you with your health?

Learning Difficulties Screening (Presence or Absence):
23. Dyslexia
24. Dysgraphia
25. Dyscalculia
26. Auditory Processing Disorder
27. Language Processing Disorder
28. Nonverbal Learning Disabilities
29. Visual Perceptual/Visual Motor Deficit

Service Usage Frequency (Scale: 1 = Very rarely to 5 = Very often):
30. I use my university's library services
31. I use the academic portal for registration and attendance updates
32. I use the Learning Management System (Moodle) offered by the university
33. I use databases offered by my university (e.g., Saudi Digital Library, Amboss, AccessPharmacy, books, and journals)
34. I regularly attend lectures and teaching sessions
35. I attend my university's orientation
36. I take benefits from academic advising services
37. I've turned to my university's psychological assistance services
38. I use apps delivered by my university to be updated on lesson and exam calendars
39. I go to my university's canteens
40. I use my university's internal and external sports/physical fitness facilities and sports services

Perception and Use of Specific Support Services (Scale: 1 to 5):
41. I find the resources granted by my university (e.g., one-on-one meetings, study tutors, electronic books) useful
42. I think that university services including academic counseling and psychological support are useful
43. I use dispensatory measures during lessons and/or exams (e.g., splitting lecture material, breaking exams into several quizzes, getting additional time to complete the exam)
44. I use technology compensatory tools during lessons and/or exams (e.g., digital recorder, calculator, technology assistance such as a reading pen)
