# Supplementary material for: University service utilization patterns in students with specific learning disabilities: An institutional cross-sectional study
Source: PLoS One. 2025 Jul 16;20(7):e0328350. doi: 10.1371/journal.pone.0328350 (PMC12266441; doi:10.1371/journal.pone.0328350)
Supplement: Attachment — Submitted filename: Response to the comments_Submission.pdf [file pone.0328350.s005.pdf]

Response to the comments

Comment 1: Please complete all items on the Human Participants Research Checklist that are relevant for your submission, by following this link: http://journals.plos.org/plosone/s/file?id=dc11/PLOSOne_Human_Subjects_Research_Checklist.docx (Contact us at plosone@plos.org if you cannot access the document.) There may be overlap between the checklist items and other queries listed below; please address any duplicated queries both in your response email and on the checklist itself. Upload the completed Human Participants Research Checklist as file type "Other" when you re-submit your manuscript. This document is for internal journal use only and will not be published if your article is accepted. The requested information will help us to assess whether your submission complies with PLOS ONE's policies and adheres to applicable reporting standards. Note that your manuscript may be rejected if you provide incomplete or inadequate responses to the checklist questions and that changing the

'Section/Category' of your article does not affect this requirement.

Response: Thanks for this comment.

We have filled in the Human Participants Research checklist, and it is being submitted along with the updated manuscript along with the IRB approval letter.

Comment 2: Please provide additional details regarding participant consent. In the ethics statement in the Methods and online submission information, please ensure that you have specified (1) whether consent was informed and (2) what type you obtained (for instance, written or verbal, and if verbal, how it was documented and witnessed). If your study included minors, state whether you obtained consent from parents or guardians. If the need for consent was waived by the ethics committee, please include this information.

Response: The following statement is now included in the methods section to clarify the ethical considerations maintained in the study.

The study was approved by the Institutional Review Board (IRB) of AlMaarefa University (IRB24-21) and adhered to STROBE guidelines for observational research. Oral informed consent was obtained from all participants, who were assured of the confidentiality and anonymity of their responses. Participation was voluntary, and participants could withdraw at any time without penalty. Participants were initially informed about the study's purpose, hypothesis, and expected outcomes. Those who provided consent accessed the online questionnaire via a QR code. The study's purpose was reiterated at the beginning of the questionnaire, and only consenting participants proceeded with the survey. Ethical standards were strictly maintained throughout the study.
