# Supplementary material for: University service utilization patterns in students with specific learning disabilities: An institutional cross-sectional study
Source: PLoS One. 2025 Jul 16;20(7):e0328350. doi: 10.1371/journal.pone.0328350 (PMC12266441; doi:10.1371/journal.pone.0328350)
Supplement: Attachment — Submitted filename: Reviewer_Comment-prof mohsin kazi.docx [file pone.0328350.s004.docx]

### **Reviewer’s Comments on the Manuscript**

#### **General Assessment:**

This study addresses a critical and timely issue—service utilization among university students with specific learning disabilities (SLDs). While the cross-sectional design offers valuable preliminary insights, certain methodological and conceptual aspects require refinement to enhance clarity, rigor, and impact. Below are detailed recommendations for improvement.

### **Abstract:**

1. **Sample and Recruitment:**
   - Specify the sample size and recruitment method (e.g., random sampling, convenience sampling).
   - Clarify whether SLDs were self-reported or clinically diagnosed, as this significantly affects validity.
2. **Interventions:**
   - The conclusion should explicitly recommend targeted interventions (e.g., awareness campaigns, tailored accommodations, faculty training) rather than broadly stating "improved support."
3. **Statistical Presentation:**
   - Ensure P (for probability) is italicized throughout the manuscript.
   - Briefly mention any validation performed for the study tool (e.g., pilot testing, Cronbach’s alpha).

### **Introduction:**

1. **Local Context:**
   - The introduction focuses heavily on SLDs in the US (Lines 79–86) but lacks context about the study’s regional setting (e.g., Middle East, specific country). Incorporate local data on SLD prevalence and university support services to strengthen relevance.
2. **University Services and Gaps:**
   - Describe common and specific services available in regional universities, citing recent literature.
   - Explicitly state the knowledge gap (e.g., "Few studies in [Region] have examined...") and how this study addresses it.

### **Methods:**

1. **Section Title:**
   - Rename "Materials and Methods" to "Subjects and Methods" for accuracy.
2. **Single-Center Study:**
   - Since this is a single-center study, the title should reflect this (e.g., "…among University Students at [Institution]").
3. **Questionnaire Validation:**
   - While Cronbach’s alpha (0.859) is reported, detail the steps in questionnaire development (e.g., expert review, factor analysis) and attach the full questionnaire as a supplementary file for reproducibility.

### **Results:**

1. **Statistical Consistency:**
   - Italicize P-values consistently in text, tables, and figures.
   - Report all significant predictors with full statistics: odds ratio (OR), 95% confidence interval (CI), and P-value (e.g., "OR 2.68, 95% CI 1.03–7.01, P = 0.044").
2. **Table & Figure Revisions:**
   - Expand all abbreviations in table legends (e.g., "cGPA: cumulative grade point average").
   - Replace ambiguous symbols (e.g., "+") with standardized notation.
   - Ensure bolded values (indicating significance) are used uniformly across tables.
   - Check all figures for standard errors addition.
   - Correct inconsistencies (e.g., Table 1 uses "*" for cGPA but legend uses "a").
3. **Interpretation of Findings:**
   - Discuss why students with dyslexia/auditory processing disorder used specific services more (e.g., "This may reflect targeted accommodations like extended exam time or assistive technologies").

### **Discussion:**

1. **Substantiate Claims:**
   - Line 443: Clarify the incomplete reference ("As reported in [19], ...").
   - Line 496: Support the claim about gender differences in help-seeking behavior with references.
2. **Specialization-Specific Analysis:**
   - Add a paragraph discussing how students’ academic specializations may influence service utilization (e.g., STEM vs. humanities).
3. **Structural Improvements:**
   - Include a dedicated "Strengths" subsection (e.g., large sample, validated tool) before limitations.
   - Consolidate recommendations into a standalone subsection (e.g., "Policy and Practical Implications").
4. **Language Refinement:**
   - Avoid overusing "underscores"; substitute with alternatives like "highlights," "emphasizes," or "demonstrates."

### **Final Recommendations:**

- **Methodological Transparency:** Disclose limitations (e.g., cross-sectional design, self-report bias).
- **Intervention Specificity:** Propose actionable strategies (e.g., "Universities should implement mandatory SLD awareness modules for faculty").
- **Reproducibility:** Share the questionnaire and detailed validation steps.

This study has significant potential to inform inclusive education policies. With these revisions, it will achieve greater scholarly impact.

**Overall Rating:** **Good (with revisions recommended for methodological and presentation clarity).**
