# Supplementary material for: University service utilization patterns in students with specific learning disabilities: An institutional cross-sectional study
Source: PLoS One. 2025 Jul 16;20(7):e0328350. doi: 10.1371/journal.pone.0328350 (PMC12266441; doi:10.1371/journal.pone.0328350)
Supplement: Attachment — Submitted filename: Response to the comments_Submission.docx [file pone.0328350.s006.docx]

Response to the editor

Journal Requirements:

When submitting your revision, we need you to address these additional requirements.

1. Please ensure that your manuscript meets PLOS ONE's style requirements, including those for file naming. The PLOS ONE style templates can be found at

<https://journals.plos.org/plosone/s/file?id=wjVg/PLOSOne_formatting_sample_main_body.pdf> and

[https://journals.plos.org/plosone/s/file?id=ba62/PLOSOne_formatting_sample_title_authors_affiliations.pdf](https://track.editorialmanager.com/CL0/https:%2F%2Fjournals.plos.org%2Fplosone%2Fs%2Ffile%3Fid=ba62%2FPLOSOne_formatting_sample_title_authors_affiliations.pdf/1/010f01977d5e983d-b84e1deb-64f7-4356-82d6-ab8696d65127-000000/4xRaEma80a1ItSlhSPmXERg0kkPdiyFva4NbIlgSpBY=215).

Response: Thanks for your recommendation.

We have updated the manuscript and formatted it as per PLOS ONE’s style requirements.

2. In the ethics statement in the Methods, you have specified that verbal consent was obtained. Please provide additional details regarding how this consent was documented and witnessed, and state whether this was approved by the IRB.

Response: Dear Editor,

Thank you for your valuable feedback regarding the ethical considerations in our study. We appreciate the opportunity to provide additional clarification about our consent procedures. Please find below our detailed response:

1. **IRB Approval:**
   - Our IRB granted explicit approval for the use of oral consent in this study, considering:
     - The minimal risk nature of the research
     - The need to protect participant anonymity
     - The practical challenges of obtaining written consent in our study design
   - The full oral consent procedure, including the script and documentation method, was reviewed and approved by the IRB.
2. **Consent Process Details:**
   - Participants received a standardized verbal explanation covering:
     - Study purpose and procedures
     - Voluntary nature of participation
     - Rights to withdraw without penalty
     - Confidentiality protections
   - Consent was reconfirmed at the questionnaire stage, with participants required to actively proceed past the consent information page.

We have revised the Methods section to include these additional details and believe these clarifications address your concerns about consent documentation and IRB approval.

3. We note that your Data Availability Statement is currently as follows: [All relevant data are within the manuscript and its Supporting Information files.]

Please confirm at this time whether or not your submission contains all raw data required to replicate the results of your study. Authors must share the “minimal data set” for their submission. PLOS defines the minimal data set to consist of the data required to replicate all study findings reported in the article, as well as related metadata and methods (<https://journals.plos.org/plosone/s/data-availability#loc-minimal-data-set-definition>).

For example, authors should submit the following data:

- The values behind the means, standard deviations and other measures reported;

- The values used to build graphs;

- The points extracted from images for analysis.

Authors do not need to submit their entire data set if only a portion of the data was used in the reported study.

If your submission does not contain these data, please either upload them as Supporting Information files or deposit them to a stable, public repository and provide us with the relevant URLs, DOIs, or accession numbers. For a list of recommended repositories, please see <https://journals.plos.org/plosone/s/recommended-repositories>.

If there are ethical or legal restrictions on sharing a de-identified data set, please explain them in detail (e.g., data contain potentially sensitive information, data are owned by a third-party organization, etc.) and who has imposed them (e.g., an ethics committee). Please also provide contact information for a data access committee, ethics committee, or other institutional body to which data requests may be sent. If data are owned by a third party, please indicate how others may request data access.

Response: We are including the data that is used for the analysis as a supplementary file 3.

4. Thank you for stating the following in the Acknowledgments Section of your manuscript:

“The authors extend their appreciation to the King Salman Center for Disability Research for funding this work through Research Group no KSRG-2024-027.”

We note that you have provided additional information within the Acknowledgements Section that is not currently declared in your Funding Statement. Please note that funding information should not appear in the Acknowledgments section or other areas of your manuscript. We will only publish funding information present in the Funding Statement section of the online submission form.

Please remove any funding-related text from the manuscript and let us know how you would like to update your Funding Statement. Currently, your Funding Statement reads as follows:

“SMBA received grant from King Salman center For Disability Research for this work through Research Group no KSRG-2024-027.”

Please include your amended statements within your cover letter; we will change the online submission form on your behalf.

Response: Please note the funding agency has the mandate to include the funding statement both in the acknowledgement section and funding section. This statement has to be included in both sections.

The authors extend their appreciation to the King Salman Center for Disability Research for funding this work through Research Group no KSRG-2024-027.

5. Please review your reference list to ensure that it is complete and correct. If you have cited papers that have been retracted, please include the rationale for doing so in the manuscript text, or remove these references and replace them with relevant current references. Any changes to the reference list should be mentioned in the rebuttal letter that accompanies your revised manuscript. If you need to cite a retracted article, indicate the article’s retracted status in the References list and also include a citation and full reference for the retraction notice.

Response: We have checked all references, and none of them were retracted from articles.

Reviewers' comments:

Reviewer #1: Dear authors,

- Overall: strong article, you're addressing a critical gap in higher education research. Holistic approach to examine both, “common” and “specific” services, providing a nuanced look at which resources students use and where improvements may be needed

Response: We sincerely thank the reviewer for their thoughtful recognition of our work’s intent. We are deeply encouraged by their appreciation of the article’s holistic approach to examining both *common* and *specific* student services. As noted, bridging this dual perspective was central to our goal of identifying not only where resources are underutilized, but also where strategic improvements could make the most meaningful impact on student success. We are grateful for the reviewer’s validation of this nuanced framework as a contribution to higher education research.

- Feedback:
a) Definition/Assessment of SLD: Relies on self-report rather than official (while also GEO dependent) diagnostic criteria or validated instruments. You might consider explaining how students identified their SLD and acknowledging that self-report may differ from formal diagnoses.

Response: We thank the reviewer for this important observation. We acknowledge that SLD identification through self-report has limitations compared to clinical diagnosis. Our approach was intentionally inclusive to capture both formally diagnosed students and those experiencing undiagnosed learning challenges – a population often underrepresented in institutional data. We have now clarified this methodology in the revised Methods section (lines 151-153) and added a dedicated limitation subsection (lines 525-533) discussing potential biases and geographic variability in diagnostic standards. While we recognize self-report may not reflect clinical diagnoses, this approach aligns with our goal of identifying students perceiving learning barriers, which directly influences their service utilization patterns.

b) Cross-Sectional Design: While capturing correlations you'll have a hard time establish causal relationships. I recommend emphasizing these design limitations in your Discussion.

Response: We thank the reviewer for this important methodological note. We fully agree that cross-sectional data cannot establish causality, and we have now explicitly emphasized this limitation in the Discussion (lines 519-525). Specifically, we:

1. Clarified that correlations **do not imply directionality** (e.g., whether distress prompts service use or vice versa),
2. Strengthened the call for **longitudinal designs** to unpack temporal relationships.

While our design was optimal for mapping *contemporary* service usage patterns across diverse student groups—addressing our primary research aim—we acknowledge that causal mechanisms require further investigation. We hope our findings provide a foundation for such future work.

c) Sampling Strategy: The recruitment process (e.g., invitations, announcements) needs more detail to assess representativeness. While you're mentioning random sampling, large parts feel like convenience sampling. Please clarify potential response biases (e.g., if certain student groups were more likely to participate).

Response: We thank the reviewer for highlighting the need for greater methodological transparency. We have revised the *Sample Recruitment* subsection (lines 204-212) to:

1. **Clarify the hybrid nature** of our approach: random *selection* but voluntary *participation*,
2. **Quantify and address potential biases**:
   - Added participation rate (302/340 = 89%)
   - Compared sample demographics to institutional data
   - Explicitly noted underrepresented groups

While random selection minimized initial sampling bias, we agree that response patterns may affect generalizability. These limitations are now explicitly discussed to support accurate interpretation.

d) Instrumentation and Reliability: Only one overall Cronbach’s alpha reported; subscale reliabilities would improve clarity and shed light on specifics. Please provide definitions or at least examples for response categories (e.g., “rarely,” “often”) to ensure consistent interpretation and minimize interpretation possibilities, as they're quite broad.

Response: We sincerely thank the reviewer for these vital methodological suggestions. We have implemented the following revisions:

1. **Subscale Reliabilities Reported**
   Added to Methods: Beyond the overall α = 0.859, subscale reliabilities are:
   - Common Services Utilization: α = 0.835 (line 162)
   - Specific Services Experience: α = 0.820 (line 169)
   - Specific Learning disabilities: α = 0.793 (line 155)
2. **Response Category Definitions**
   » Added temporal anchors: Frequency categories were defined as:
   1 (Very rarely) = ≤1x/semester; 2 (Rarely) = 2-3x/semester; 3 (Sometimes) = monthly; 4 (Often) = bi-weekly; 5 (Very often) = weekly+
3. **Full Instrument Accessibility**
   Added disclosure: As recommended by Reviewer #2, the complete questionnaire, including all items, and response categories is provided as **Supplemental File 1** (line 141)

e) Statistical Reporting: While the use of chi-square tests and multinomial regressions is appropriate, I'd like to see greater transparency. For instance, if you performed multiple comparisons, please clarify whether any p-value adjustments were made (like Bonferroni). Also, please justify the choice of multinomial over simpler logistic models. Additionally, consider reporting more precise effect sizes—such as Cohen’s d or odds ratios with full confidence intervals—in the tables for clarity.

Response: We acknowledge that no p-value adjustments (e.g., Bonferroni) were applied, as our analysis was primarily exploratory and hypothesis-generating for service utilization patterns. To maximize sensitivity in detecting potential associations—common in early-stage educational research—we reported uncorrected p-values with explicit 95% confidence intervals for all results.

We sincerely thank the reviewer for highlighting this critical clarification. We confirm the service utilization outcome is strictly binary (categorized as *Rarely* or *Often*), not multinomial as previously misstated. Consequently, we have replaced all multinomial regression analyses with binary logistic regression—a statistically appropriate method aligned with our dichotomous outcome variable—to ensure accurate modeling of predictors influencing service engagement frequency.

Comprehensive outputs for all regression analyses—including unstandardized coefficients (B), standard errors (S.E.), Wald statistics, degrees of freedom (df), p-values (Sig.), odds ratios (Exp(B)), and 95% confidence intervals for odds ratios—are provided in Supplementary File 2 for common services and Supplementary File 5 for specific services."

f) Detail on Service Usage: “Common” vs. “specific” categories are helpful to grasp the big picture but may obscure usage differences for individual services. You could highlight which specific resources are used most/least to pinpoint where support might be lacking.

Response: Thanks for your valuable recommendation. We have now extensively discussed the types of facilities under both common and specific domains, with the inclusion of two figures (Figs 5 and 6).

g) Interpretation of SLD Subtypes: You could offer more context on how each SLD subtype might explain different usage rates and tie the nature of each SLD to potential barriers in accessing or benefiting from certain services.

Response: We appreciate your recommendation. We have now extensively compared types of common and specific university services with the types of SLDs, and Tables 4 and 5 are added. Subsequently, the numbering of other tables is re-sequenced.

h) Documentation of Oral Consent: While you state that oral informed consent was obtained, without having access to the files it remains unclear how exactly you documented participants’ agreement in the online setting. Please clarify especially whether participants, for example, had to check a digital box or confirm an introductory text or audio file before proceeding, so readers understand how consent was explicitly provided and recorded.

Response: We appreciate the opportunity to provide additional clarification about our consent procedures. Please find below our detailed response:

1. **Consent Process Details:**
   - Participants received a standardized verbal explanation covering:
     - Study purpose and procedures
     - Voluntary nature of participation
     - Rights to withdraw without penalty
     - Confidentiality protections
   - Consent was reconfirmed at the questionnaire stage, with participants required to actively proceed past the consent information page (this was included as first page in the online version of the questionnaire); those who consent will proceed to participate in recording their feedback).

We have revised the Methods section to include these additional details about consent (line 222-232).

Reviewer #2: Reviewer’s Comments on the Manuscript
General Assessment:
This study addresses a critical and timely issue—service utilization among university students with specific learning disabilities (SLDs). While the cross-sectional design offers valuable preliminary insights, certain methodological and conceptual aspects require refinement to enhance clarity, rigor, and impact. Below are detailed recommendations for improvement.

Response: We appreciate your insightful evaluation of the manuscript.

Abstract:
1. Sample and Recruitment:
o Specify the sample size and recruitment method (e.g., random sampling, convenience sampling).
o Clarify whether SLDs were self-reported or clinically diagnosed, as this significantly affects validity.
2. Interventions:
o The conclusion should explicitly recommend targeted interventions (e.g., awareness campaigns, tailored accommodations, faculty training) rather than broadly stating "improved support."
3. Statistical Presentation:
o Ensure P (for probability) is italicized throughout the manuscript.
o Briefly mention any validation performed for the study tool (e.g., pilot testing, Cronbach’s alpha).

Response: Thanks for your valuable comments.

1. **Sample and Recruitment:**
   - **Sample size:** Added (*n* = 302).
   - **Recruitment method:** Specified as "random but voluntary sampling".
   - **SLD ascertainment:** Clarified as "self-reported specific learning disabilities (SLDs)" throughout.
2. **Interventions:**
   - Revised the conclusion to explicitly recommend **targeted interventions**: "disability-awareness campaigns", "tailored accommodations", and "mandatory faculty training" (replacing broad statements).
3. **Statistical Presentation:**
   - Ensured *P* (probability) is consistently *italicized* (e.g., *P*<.001, *P*=0.024).
   - Added tool validation: "using a tool demonstrating good internal reliability (Cronbach’s α = 0.859)".

Introduction:
1. Local Context:
o The introduction focuses heavily on SLDs in the US (Lines 79–86) but lacks context about the study’s regional setting (e.g., Middle East, specific country). Incorporate local data on SLD prevalence and university support services to strengthen relevance.

Response: We are thankful for this valuable recommendation.

In response to the comments of the respected reviewer, we have made the updates in the Introduction section with the inclusion of data from Saudi Arabian context and availability of support services (Line 129-145).

2. University Services and Gaps:
o Describe common and specific services available in regional universities, citing recent literature.
o Explicitly state the knowledge gap (e.g., "Few studies in [Region] have examined...") and how this study addresses it.

Response: We appreciate the important comment of the respected reviewer.

We have updated the introduction section with inclusion of the latest findings about the services offered in the universities and gaps in those facilities (Line 101-128).

Methods:
1. Section Title:
o Rename "Materials and Methods" to "Subjects and Methods" for accuracy.
2. Single-Center Study:
o Since this is a single-center study, the title should reflect this (e.g., "…among University Students at [Institution]").
3. Questionnaire Validation:
o While Cronbach’s alpha (0.859) is reported, detail the steps in questionnaire development (e.g., expert review, factor analysis) and attach the full questionnaire as a supplementary file for reproducibility.

Response:

1. **Section Title Renaming:**

The section "Materials and Methods" has been renamed **"Subjects and Methods"** throughout the manuscript to accurately reflect the study design (Page 3, Line 110).

2. **Single-Center Study Declaration in Title:**

The title has been revised to **"University Service Utilization Patterns in Students with Specific Learning Disabilities: An Institutional Cross-Sectional Study"** to explicitly indicate the single-university scope (Page 1, Line 5).

3. **Questionnaire Validation Enhancements:**

**Development Process:** The questionnaire underwent **expert review** (3 content specialists) to establish construct validity. The questionnaire was pilot-tested with 20 participants randomly selected from the eligible student population to evaluate clarity, feasibility, and item comprehension. Feedback was incorporated to refine wording and structure, resulting in an instrument demonstrating excellent internal consistency.

**Reliability Metrics:**

- Overall instrument: **Cronbach’s α = 0.859**
- Subscale reliabilities added:
  - Specific Learning Disabilities: **α = 0.793** (Line 155)
  - Common Services Utilization: **α = 0.835** (Line 162)
  - Specific Services Experience: **α = 0.820** (Line 169)

**Reproducibility:** The full questionnaire is provided as **Supplementary File 1**.

Results:
1. Statistical Consistency:
o Italicize P-values consistently in text, tables, and figures.
o Report all significant predictors with full statistics: odds ratio (OR), 95% confidence interval (CI), and P-value (e.g., "OR 2.68, 95% CI 1.03–7.01, P = 0.044").
2. Table & Figure Revisions:
o Expand all abbreviations in table legends (e.g., "cGPA: cumulative grade point average").
o Replace ambiguous symbols (e.g., "+") with standardized notation.
o Ensure bolded values (indicating significance) are used uniformly across tables.
o Check all figures for standard errors addition.
o Correct inconsistencies (e.g., Table 1 uses "*" for cGPA but legend uses "a").
3. Interpretation of Findings:
o Discuss why students with dyslexia/auditory processing disorder used specific services more (e.g., "This may reflect targeted accommodations like extended exam time or assistive technologies").

Response: We are grateful for the valuable comments of the reviewer. Based on the recommendations, the following changes have been made in the manuscript:

1. P values are consistently presented in italics throughout the manuscript.
2. We have included aOR in all places where regression analysis is discussed.
3. All abbreviations are expanded and included in the legend.
4. Ambiguous symbols are replaced.
5. Consistency is maintained for symbols used in the table and denoted in the legend.
6. Findings are interpreted in the discussion section.
7. Tables 5 and 6 are added to demonstrate the utilization of common and specific services, respectively. Data is given in mean±SEM, while data in Fi 1-4 are given in percentage that cannot be presented with SEM.

Discussion:
1. Substantiate Claims:
o Line 443: Clarify the incomplete reference ("As reported in [19], ...").
o Line 496: Support the claim about gender differences in help-seeking behavior with references.
2. Specialization-Specific Analysis:
o Add a paragraph discussing how students’ academic specializations may influence service utilization (e.g., STEM vs. humanities).
3. Structural Improvements:
o Include a dedicated "Strengths" subsection (e.g., large sample, validated tool) before limitations.
o Consolidate recommendations into a standalone subsection (e.g., "Policy and Practical Implications").
4. Language Refinement:
o Avoid overusing "underscores"; substitute with alternatives like "highlights," "emphasizes," or "demonstrates."

Response: We are thankful to the respected reviewers for their insightful comments. We have comprehensively updated the discussion section to address each comment of the reviewer. Line 526-681.

Final Recommendations:
• Methodological Transparency: Disclose limitations (e.g., cross-sectional design, self-report bias).
• Intervention Specificity: Propose actionable strategies (e.g., "Universities should implement mandatory SLD awareness modules for faculty").
• Reproducibility: Share the questionnaire and detailed validation steps.
This study has significant potential to inform inclusive education policies. With these revisions, it will achieve greater scholarly impact.

Response: We appreciate the valuable comment and observation of the reviewer. We have included the questionnaire as supplementary file 1.
